# A Hybrid Approach to Protect Palmprint Templates

**DOI:** 10.1155/2014/686754

**Published:** 2014-03-27

**Authors:** Hailun Liu, Dongmei Sun, Ke Xiong, Zhengding Qiu

**Affiliations:** ^1^School of Computer & Information Technology, Beijing Jiaotong University, Beijing 100044, China; ^2^Beijing Key Laboratory of Advanced Information Science and Network Technology, Beijing 100044, China

## Abstract

Biometric template protection is indispensable to protect personal privacy in large-scale deployment of biometric systems. Accuracy, changeability, and security are three critical requirements for template protection algorithms. However, existing template protection algorithms cannot satisfy all these requirements well. In this paper, we propose a hybrid approach that combines random projection and fuzzy vault to improve the performances at these three points. Heterogeneous space is designed for combining random projection and fuzzy vault properly in the hybrid scheme. New chaff point generation method is also proposed to enhance the security of the heterogeneous vault. Theoretical analyses of proposed hybrid approach in terms of accuracy, changeability, and security are given in this paper. Palmprint database based experimental results well support the theoretical analyses and demonstrate the effectiveness of proposed hybrid approach.

## 1. Introduction

Biometric based authentication is more convenient and reliable than password or token based authentication. However, biometric technology needs large-scale capture and storage of biometric data which leads to serious concern about privacy leakage and identity theft. Unlike passwords or tokens, biometric characteristics are inherent to a person; once they are compromised, they would never be reissued or refreshed. Therefore, biometric template protection techniques [1] have attracted much attention recently for the reasons mentioned above.

Broadly, biometric template protection techniques can be categorized into two classes, cancelable biometrics and biometric cryptosystems. For a typical biometric template protection scheme, three critical requirements are suggested to satisfy [2]. 

*(1) Accuracy Requirement*. The discriminability of original biometric features should be preserved in a biometric template protection scheme, so that the accuracy of biometric system is not degraded.

*(2) Security Requirement*. The protected objects (biometric features and cryptographic key in biometric cryptosystems) should be computationally hard to be revealed by attackers even though the sketch is published.

*(3) Cancelability Requirement*. The cancelability means revocability and diversity. Different applications have different templates of the same user, and these templates cannot authenticate with each other. Once a template is compromised, a new different template can be generated to replace it.

However, for cancelable biometrics and biometric cryptosystems, they cannot satisfy all these requirements quite well. And different approach has its advantages and disadvantages [3].

The cancelable biometrics often uses transform-based approach to generate new templates. This approach has good cancelability, but the security level is often lower than biometric cryptosystems, and in general no independent cryptographic key can be bound for cryptographic applications.

Biometric cryptosystems (BC) [4] output encrypted sketch; the security level is relatively high. BC uses biometric features to protect cryptographic key, which provide a new solution for key management issue. However, the error correcting code (ECC) used in this technique is not strong enough to handle large biometric intraclass variants; the accuracy of BC degrade sharply, and changeability is often not provided.

Considering the limitations of available approaches, hybrid approach [5, 6] is a solution to meet the increasing demands for biometric template protection.

In this paper, a novel hybrid approach is proposed to compensate the shortcomings of a single approach and meanwhile maintain the advantages of individual approach in the hybrid scheme.

The proposed hybrid scheme combines fuzzy vault scheme (FVS) [7] and random projection [8] to meet above three requirements for biometric template protection.

Fuzzy vault scheme is one of the most popular biometric cryptosystems [9–11]; it provides an effective security mechanism to protect cryptographic key and biometric templates simultaneously. However, the system accuracy in terms of false accept rate (FAR) and false rejection rate (FRR) often degrade sharply due to insufficient intraclass variations handing ability of used error correcting code. And FVS do not provide cancelability. The random projection, which is a transform-based template protection approach, has good cancelability property. By combining the random projection method with the fuzzy vault scheme, the proposed hybrid scheme aims to improve the accuracy and security and provide good changeability simultaneously.

To combine random projection with fuzzy vault effectively, first, a heterogeneous space is defined; raw biometric features are projected into the heterogeneous space by random projection and long enough cryptographic key can be bound together with projected features in the heterogeneous space. A new chaff point generation method is also proposed to ensure the security even when the projection matrices are lost, and then three requirements of proposed hybrid are theoretically analyzed. Promising experimental results based on palmprint database show the validity of proposed hybrid approach.

The rest of this paper is organized as follows. The proposed hybrid approach is described in Section 2. Three requirements are analyzed in Section 3. Experimental results are reported in Section 4. All works are summarized in Section 5.

## 2. Proposed Hybrid Approach

**Figure 1:**
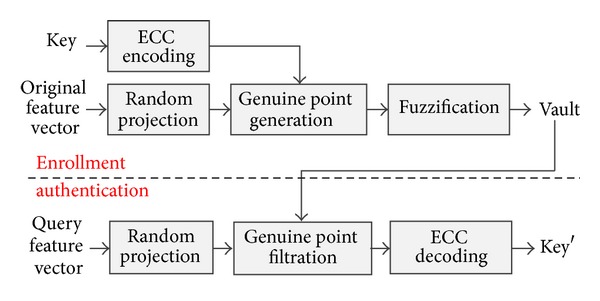
Flow chart of the proposed hybrid algorithm.

The flow chart of proposed hybrid approach is shown in Figure 1, in which two main modules are included. The first is multispace random projection which is used not only to provide cancelability but also to provide the different representations of original palmprint feature vectors in random subspaces for generating different genuine points. The second is the proposed heterogeneous fuzzy vault scheme, which is used to enhance security and bind cryptographic key for cryptographic applications. Since cryptographic key is generated independently, its randomness is guaranteed, and in heterogeneous space, the cryptographic key can be bound long enough to meet high security requirements in cryptographic applications. In the following subsections, we will introduce how these two modules work.

### 2.1. Multispace Random Projection

Assuming the fixed-length feature vector is **x** ∈ *ℜ* ^*n*^, the multispace random projection is defined as follows [8]:

(1) $$\begin{array}{c} v=\sqrt{\frac{1}{m}}R^{T}x, \end{array}$$

where *R* is a random matrix with size *n* × *m* and *T* represents matrix transposition.

In order to generate multiple genuine points using single feature vector, one feature vector **x** ∈ *ℜ* ^*n*^ is projected into a set of random subspaces by using different projection matrices:

(2) $$\begin{array}{c} v_{i}=\sqrt{\frac{1}{m}}R_{i}^{T}x,\;\text{where}\;\;i=1,\ldots ,g. \end{array}$$

### 2.2. Generation of Heterogeneous Vault

The heterogeneous vault is a set of points in* heterogeneous space*. The* heterogeneous space* is defined as {*v* ∈ *ℜ* ^*m*^, *s* ∈ *F*_*q*_}, where *v* ∈ *ℜ* ^*m*^ is a real-valued vector and *m* is its length; *s* ∈ *F*_*q*_ is an element from finite field *F*_*q*_, where *q* is the cardinality of the finite field. A heterogeneous vault contains two subsets, genuine points and chaff points. Following we will introduce how to generate these two parts.

#### 2.2.1. Generation of Genuine Points

*(a) Feature Vector Mapping. *We have

(3) $$\begin{array}{c} x\in {ℜ}^{n}⟶{\left\{v_{i}\in {ℜ}^{m}\right\}}_{i=1}^{t}. \end{array}$$

The high dimensional palmprint feature vector **x** ∈ *ℜ* ^*n*^ is mapped into *t* low dimensional subvectors using (2); that is, $v_{i}=\sqrt{1/m}R_{i}^{T}x$. *v*_*i*_ is named genuine vector.

In genuine vector generation, *t* projection matrices *R*_*i*_ are used on one original feature vector *x* to generate *t* different genuine vectors.

*(b) Key Encoding. *We have

(4) $$\begin{array}{c} \kappa ⟶{\left\{s_{i}\in F_{q}\right\}}_{i=1}^{t}. \end{array}$$

The key *κ* to be protected is independent of genuine vectors, so that it can be generated randomly; therefore, the randomness of the key is guaranteed.

In this step, the key *κ* to be protected is encoded into *t*-symbol sequence {*s*_*i*_ ∈ *F*_*q*_}_*i*=1_^*t*^ using ECC encoding algorithm. If the key is very long, it can be segmented into multiple shorter sequences, and then each shorter sequence is encoded into *t*-symbol sequence; that is, {{*s*_*ji*_∈*F*_*q*_}_*i*=1_^*t*^}_*j*=1_^*N*^, where *N* is the number of segmented sequences.

*(c) Pairwise Conjugation. *We have

(5) $$\begin{array}{c} {\left\{v_{i}\in {ℜ}^{m}\right\}}_{i=1}^{t}+{\left\{s_{i}\in F_{q}\right\}}_{i=1}^{t}⟶{\left\{v_{i}\in {ℜ}^{m},s_{i}\in F_{q}\right\}}_{i=1}^{t}. \end{array}$$

Given genuine vector {*v*_*i*_ ∈ *ℜ* ^*m*^}_*i*=1_^*t*^ obtained in step (a) and *t*-symbol sequence {*s*_*i*_ ∈ *F*_*q*_}_*i*=1_^*t*^ obtained in step (b), *t* genuine points {*v*_*i*_ ∈ *ℜ* ^*m*^, *s*_*i*_ ∈ *F*_*q*_}_*i*=1_^*t*^ belong to heterogeneous space can be generated by combining genuine vectors and symbols orderly. If longer key needs to be bound, each genuine vector can be combined with multiple symbols, that is, {*v*_*i*_ ∈ *ℜ* ^*m*^, *s*_1*i*_, *s*_2*i*_,…, *s*_*Ni*_ ∈ *F*_*q*_}_*i*=1_^*t*^. For the pairwise conjugation, in vault unlocking, the recognition errors of genuine vectors are transformed to symbol errors in the *t*-symbol sequence, so that can be corrected by the ECC decoding algorithm.

#### 2.2.2. Generation of Chaff Points

The chaff points are generated to protect genuine points against attacks such as clustering attack and compromised projection matrices attack.

The chaff points {cv_*j*_ ∈ *ℜ* ^*m*^, cs_*j*_ ∈ *F*_*q*_}_*j*=1_^*r*−*t*^ have the same components as genuine points; that is, chaff vector cv_*j*_ ∈ *ℜ* ^*m*^ and chaff symbol cs_*j*_ ∈ *F*_*q*_. Since secret symbols *s*_*i*_ in genuine points are generated randomly, the chaff symbols cs_*j*_ can be selected randomly from Galois field *F*_*q*_.

**Figure 2:**
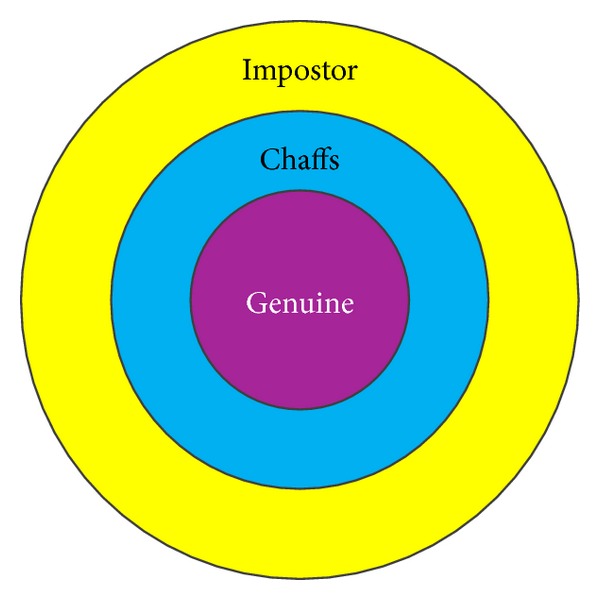
Illustration of chaff point generation idea.

The idea of chaff vector generation is shown in Figure 2, where genuine matching distances are concentrated in the smallest circle, impostor matching distances are in the largest circle, and chaff vectors are added in the middle circle, so as to prevent the adversary from knowing which are genuine vectors, even though the adversary has impostor biometric features.

The chaff vectors cv_*j*_ are generated as follows: cv_*j*_ = *v*_*i*_ + *α* · rv_*j*_, where, *v*_*i*_ is genuine vector, and rv_*j*_ is a random vector; each element in rv_*j*_ is independent and identically distributed (i.i.d.) according to standard norm distribution *N*(0,1). Then, ||rv_*j*_||^2^ follows a chi-square distribution with degree of freedom *m*, and its expectation *E*(||rv_*j*_||^2^) = *m*. To control the distance between chaff point and genuine point, the *α* is used as a scaling factor. The value of *α* is set to be $\sqrt{t^{2}/m}$, where *t* is selected according to the genuine and imposter distributions of matching distances of projected feature vectors.

**Figure 3:**
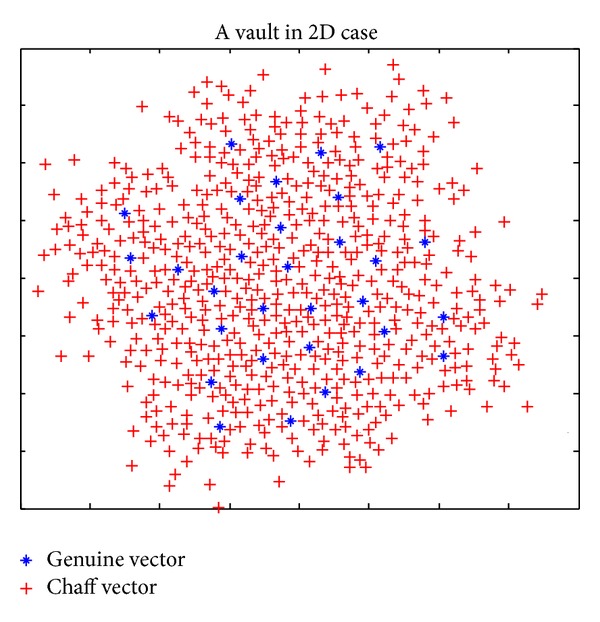
A 2D vault with genuine vectors and chaff vectors.

Although the distances between one genuine vector and its chaff vectors are concentrated around its mean *t*, the distances are distributed randomly; a small number of chaff vectors may be very close to some genuine vectors, which will lead to failure of genuine point filtration in vault decoding phase. Here, a minimum distance threshold *δ* and maximum distance threshold *λ* are set for all points in vault to reduce filtration errors and prevent attackers from recognizing chaff points by distance analysis. The minimum distance threshold *δ* is less than *t* and the maximum distance threshold *λ* is greater than *t*, the same as *t*; both *δ* and *λ* are selected according to the genuine and imposter matching distances distribution of projected feature vectors. An example of a 2D vault generated applying proposed genuine and chaff points generation methods is illustrated in Figure 3.

After adding chaff points, all points in heterogeneous space are sorted according to the value of the first elements in real-valued vectors; after that, the vault can be stored in smartcard or central database.

### 2.3. Decoding of Heterogeneous Vault

*(1) Query Subvectors Generation. *We have

(6) $$\begin{array}{c} qx\in {ℜ}^{n}⟶{\left\{{\text{qv}}_{i}\in {ℜ}^{m}\right\}}_{i=1}^{t}. \end{array}$$

Firstly, the query feature vector *qx* ∈ *ℜ* ^*n*^ is projected into query subvectors {qv_*i*_ ∈ *ℜ* ^*m*^}_*i*=1_^*t*^ using the projection matrices according to (2).

*(2) Filtration of Genuine Points by Distance Measure.* The genuine vector filtration is carried out between query subvectors {qv_*i*_ ∈ *ℜ* ^*m*^}_*i*=1_^*t*^ and the vault {*v*_*i*_ ∈ *ℜ* ^*m*^, *s*_*i*_ ∈ *F*_*q*_}_*i*=1_^*r*^. Given query subvector qv_*i*_, computing distances between qv_*i*_, and real-valued vectors *v*_*i*_ in all points in vault, the point in vault corresponding to the minimum distance is considered as the genuine point.

Totally, there are *t* points {ov_*i*_ ∈ *ℜ* ^*m*^, os_*i*_ ∈ *F*_*q*_}_*i*=1_^*t*^ that are filtered out orderly from vault, and then os_*i*_ are extracted from filtered points and cascaded orderly to form a *t*-symbol sequence {os_*i*_ ∈ *F*_*q*_}_*i*=1_^*t*^ for ECC decoding.

*(3) Correcting Error Symbols Using ECC Decoding Algorithm *{os_*i*_ ∈ *F*_*q*_}_*i*=1_^*t*^ → *κ*′. Given *t*-symbol sequence {os_*i*_ ∈ *F*_*q*_}_*i*=1_^*t*^ obtained in previous step, a proper ECC decoding algorithm is used to such sequence to get *κ*′. The false filtration of genuine points would result in symbol errors in {os_*i*_ ∈ *F*_*q*_}_*i*=1_^*t*^, and the number of error symbols equals to the number of falsely recognized genuine points. If the number of error symbols is within the error-correcting capability of ECC, the original key *κ* can be recovered successfully by ECC decoding algorithm; that is, *κ*′ = *κ*.

## 3. Analysis of Proposed Hybrid Approach

In this section, the accuracy, changeability, and security of proposed hybrid approach are analyzed theoretically.

### 3.1. Accuracy Analysis

#### 3.1.1. Nonorthogonal Matrix Case

If the projection matrices are nonorthogonal, the random projection can preserve the pairwise distances at a certain degree; this property is addressed by means of the Johnson-Lindenstrauss (JL) Lemma [2].

*J-L Lemma*. For any 0 < *ϵ* < 1 and any integer *k*, let *m* be a positive integer such that *m* ≥ *M*_0_ = *O*  (*ϵ* ^−2^log⁡*k*). Then, for any set *S* of *k* points in *ℜ* ^*n*^, there is a map *f* : *ℜ* ^*n*^ → *ℜ* ^*m*^, such that for all **x**, **y** ∈ *S*,

(7) $$\begin{array}{c} \left(1-\epsilon \right){||x-y||}^{2}⩽{||f\left(x\right)-f\left(y\right)||}^{2}⩽\left(1+\epsilon \right){||x-y||}^{2}. \end{array}$$

According to the J-L Lemma, an original set with *k* points in *n*-dimension Euclidean space can be embedded into another Euclidean space with dimension *O*(*ϵ* ^−2^log⁡*k*); meanwhile, the pairwise distances of points are preserved up to a factor of *ϵ*. Arriaga and Vempala [12], Achlioptas [13], and Li et al. [14] have proved that such mapping can be achieved by random projections.

This property states that we can change the form of real-valued biometric feature vectors, but the discriminability of feature vectors are still preserved. So, this property can be used to generate multiple genuine vectors in vault generation.

#### 3.1.2. Orthogonal Matrix Case

In this case, the projection matrix *R*_*i*_ is a square matrix; that is, *R*_*i*_ ∈ *ℜ* ^*n*×*n*^. Since each entry of *R*_*i*_ is an independent and identically distributed random variable, by applying Gram-Schmidt orthonormalization method [13], the projection matrix can be transformed to an orthogonal matrix to obtain *RR* ^*T*^ = *R* ^*T*^ *R* = *I*, where *I* is an identity matrix. In this case, the random projection becomes orthogonal transformation.

Suppose that *x*_*i*_, *x*_*j*_ ∈ *ℜ* ^*n*^ are two different real-valued feature vectors and *R* ∈ *ℜ* ^*n*×*n*^ is orthogonal matrix; then [15], we have

(8) ||RTxi−RTxj||2=(RTxi−RTxj)T(RTxi−RTxj)=(xi−xj)TRRT(xi−xj)=(xi−xj)(xi−xj)=||xi−xj||2.

The above equation demonstrates that the pairwise Euclidean distances of feature vectors can be precisely preserved after orthogonal random projection.

### 3.2. Changeability Analysis

The changeability of proposed scheme is provided by the random projection module. By refreshing the projection matrices, the projected feature vector can be updated. In this subsection, the statistical properties [16] of random projection are used for changeability analysis.

Let *u*, *v* ∈ *ℜ* ^*n*^ be two feature vectors of the same user; *R*, *S* ∈ *ℜ* ^*n*×*m*^, *m* ≤ *n*, are two different random matrices, assuming that each entry of *R* or *S* follows standard normal distribution **N**(0,1); then, applying the same projection matrix for projection; that is, $x=\sqrt{1/m}R^{T}u$, $y=\sqrt{1/m}R^{T}v$, the mean and variance of squared Euclidean distance between *x* and *y* are as follows [16]:

(9) $$\begin{array}{c} E\left[{||x-y||}^{2}\right]={||u-v||}^{2}, \end{array}$$

(10) $$\begin{array}{c} \mathrm{Var}\left[{||x-y||}^{2}\right]=\frac{2}{m}{||u-v||}^{4}. \end{array}$$

According to (9), after projection, the mean of squared Euclidean distances is the same as the distance of two original feature vectors. According to (10), the variance is inversely proportional to the dimension of new space. The higher the dimension, the smaller the variance, which means better preservation of pairwise distances between original feature vectors.

If projection matrices are different; that is, $x=\sqrt{1/m}R^{T}u$, $y=\sqrt{1/m}S^{T}v$, the corresponding mean and variance are as follows [16]:

(11) $$\begin{array}{c} E\left[{||x-y||}^{2}\right]={||u||}^{2}+{||v||}^{2}, \end{array}$$

(12) $$\begin{array}{c} \mathrm{Var}\left[{||x-y||}^{2}\right]=\frac{2}{m}{\left({||u||}^{2}+{||v||}^{2}\right)}^{2}. \end{array}$$

According to (9) and (11), since ||*u*−*v*||^2^ ≤ ||*u*||^2^ + ||*v*||^2^, when different projection matrices are applied for projections, the gathering center of squared Euclidean distances of pairwise vectors in new space is larger than that in same projection matrices scenario. According to (10) and (12), larger *m* means smaller variances, which leads to clear separation of two kinds of distance distributions, so that stronger changeability can be provided.

### 3.3. Security Analysis

Assuming that an attacker has obtained the vault and all parameters of the vault, that is, the number of genuine points *t*, the number of chaff points *r* − *t*, and the number of symbol errors *k* that can be corrected in vault decoding phase, the security of the vault is considered in four different circumstances.

#### 3.3.1. The Attacker Has No Information about Projection Matrices and Impostor Features

In this condition, what an attacker can do is to employ brute force attack to decode the vault. Min-entropy [17] is used to measure the security of the vault:

(13) $$\begin{array}{c} H_{\infty }=\log\left(C_{r}^{t-k}P_{t-k}^{t-k}\right), \end{array}$$

where “*C*” means the number of combinations and “*P*” means the number of permutations.

#### 3.3.2. The Attacker Has Genuine Query Feature Vector

In this case, the attacker will use randomly generated random matrices *R*_*A*_ ∈ *ℜ* ^*n*×*m*^ and legitimate query feature vector *V*_*A*_ ∈ *ℜ* ^*m*^ to decode the vault. The security of the vault can be measured by the false accept probability *P*_*f*_.

Assuming projection matrices used in enrollment are *R*_*E*_ and *R*_*E*_ ≠ *R*_*A*_, enrolled feature vector and lost legitimate feature vectors are *V*_*E*_ and *V*_*A*_, respectively. The transformed features are $X_{E}=\sqrt{1/m}R_{E}^{T}V_{E}$ and $X_{A}=\sqrt{1/m}R_{A}^{T}V_{A}$, respectively.

Since each entry in *R*_*A*_ and *R*_*E*_ is generated randomly, they can be full column rank matrices, and therefore $\sqrt{1/m}R_{E}$ and $\sqrt{1/m}R_{A}$ can be decomposed [18] as follows: $\sqrt{1/m}R_{E}=UQ_{E}$ and $\sqrt{1/m}R_{A}=UQ_{A}$, where *U* ∈ *ℜ* ^*n*×*m*^ and *U* ^*T*^ *U* ≈ *I*. *Q*_*E*_ and *Q*_*A*_ ∈ *ℜ* ^*m*×*m*^. Since *U* ^*T*^ *U* ≈ *I*, there are $Q_{E}=\sqrt{1/m}U^{T}R_{E}$ and $Q_{A}=\sqrt{1/m}U^{T}R_{A}$, and columns of *Q*_*E*_ and *Q*_*A*_ are almost orthonormal. Then, the projected features can be reformulated as *X*_*E*_ = *Q*_*E*_^*T*^(*U* ^*T*^ *V*_*E*_) and *X*_*A*_ = *Q*_*A*_^*T*^(*U* ^*T*^ *V*_*A*_).

These two equalities imply that original feature vectors are first projected by the same matrix *U* and then transformed using different orthonormal matrices, which is equivalent to the rotation of a point in hyperspace; the rotation radius is the length (norm) of the point.

According to geometric-based analysis in [18], the false accept probabilities are obtained in two cases:

(14) $$\begin{array}{c} P_{f1}=\frac{t^{m}}{{\left(l_{XE}+t\right)}^{m}},\;\text{when}\;\;l_{XE}\leq t,\\ P_{f2}=\frac{t^{m}}{{\left(l_{XE}+t\right)}^{m}-{\left(l_{XE}-t\right)}^{m}},\;\text{when}\;\;l_{XE}>t, \end{array}$$

where *t* is a controlling threshold in chaff vector generation, *m* is the dimension of projected feature vectors, and *l*_*XE*_ and *l*_*XA*_ are length of *X*_*E*_ and *X*_*A*_, respectively.

From the above two cases, the total false accept probability can be expressed as

(15) Pf=P(lXA≤lXE+t ∣ lXE≤t)P(lXE≤t)Pf1 +P(lXE−t≤lXA≤lXE+t ∣ lXE>t)P(lXE>t)Pf2.

The total false accept probability depends on dimension *m* of projected feature vector and the threshold *t*.

#### 3.3.3. The Attacker Has the Projection Matrices

When the attacker only has projection matrices *R*_*E*_, we consider a scenario that a random vector *V*_*r*_ is generated as query feature vector; after projection, *X*_*r*_ = *R*_*E*_^*T*^ *V*_*r*_ is used to decode the vault. The probability that *X*_*r*_ falls into the hyperspace where the distance between *X*_*r*_ and a genuine vector *X*_*G*_ = *R*_*E*_^*T*^ *V*_*E*_ is less than a threshold *T* which is proposed to measure the security in this case.

Suppose Euclidean distance is used to measure the distance between two vectors; the probability can be written as follows:

(16) $$\begin{array}{c} \text{Pr}\left({||X_{r}-X_{G}||}_{2}<T\right)=\text{Pr}\left({||R_{E}^{T}\left(V_{r}-V_{E}\right)||}_{2}<T\right). \end{array}$$

Assuming that entries in *V*_*r*_ are uniformly and independently distributed in a given value range *I*, to simplify the calculation, we transform the above probability to the probability that each random generated element in *V*_*r*_ falls into a small value range; that is,

(17) $$\begin{array}{c} \text{Pr}\left({||R_{E}^{T}(V_{r}-V_{E})||}_{2}<T\right)\approx \prod_{i=1}^{n}\text{Pr}\left(V_{E}^{i}-\Delta <V_{r}^{i}<V_{E}^{i}+\Delta \right). \end{array}$$

Since uniformly distribution in a given value range *I* is assumed for entries in *V*_*r*_, the probability that each entry *V*_*r*_^*i*^ falls into the given value range 2Δ is as follows:

(18) $$\begin{array}{c} \text{Pr}\left(V_{E}^{i}-\Delta <V_{r}^{i}<V_{E}^{i}+\Delta \right)=\frac{2\Delta }{I}. \end{array}$$

Substituting (18) into (17), we get

(19) $$\begin{array}{c} \text{Pr}\left({||R_{E}^{T}(V_{r}-V_{E})||}_{2}<T\right)\approx {\left(\frac{2\Delta }{I}\right)}^{n}, \end{array}$$

where *n* is the length of *V*_*r*_.

#### 3.3.4. The Attacker Has Projection Matrices *R* and Impostor Feature Vector *V*_*I*_

This case is the user-independent scenario; all users use the same projection matrices. The attacker may take *X*_*I*_ = *R* ^*T*^ *V*_*I*_ as a center to determine a hypersphere to find genuine points. According to proposed chaff point generation method, chaff vectors are added much closer to genuine vector than query vectors projected from impostor feature vectors, even though genuine projection matrices are used. So for each genuine vector, there will be lots of chaff vectors in the hypersphere in which the attacker does not know which one is exactly the genuine vector.

From the fuzzification phase in vault generation, we know there are *t* genuine points and *r* − *t* chaff points in a vault. Averagely, there are *r*/*t* points in a hypersphere. In these *r*/*t* points, only one is genuine point. Assuming *k* symbol errors can be corrected by the ECC; then, the security of vault can be computed as follows:

(20) $$\begin{array}{c} H=\text{lo}{\text{g}}_{2}{\left(C_{r/t}^{1}\right)}^{t-k}. \end{array}$$

In the above four different scenarios, the last one is the most severe scenario since the attacker has gotten most information. In (20), there are three variables, total number of points in vault *r*, the number of genuine points in vault *t*, and the number of corrected symbols *k* by ECC. The quantified bits and the trend of security when changing different parameters will be discussed in next section.

## 4. Experimental Results and Discussion

In this section, the proposed hybrid scheme is evaluated based on palmprint database. Concrete experimental results in terms of accuracy, changeability, and security are presented to support the proposed hybrid approach.

### 4.1. Palmprint Database and Experimental Parameters

The Handmetric Authentication Beijing Jiao Tong University database (HA-BJTU) [19] is used in experiments. In HA-BJTU, there are 1973 palmprints of 98 people. The palmprints are resampled to 128 × 128, and the resolution of palmprint image is 72 dpi.

The classic principle component analysis (PCA) and linear discriminant analysis (LDA) are used to extract the features from palmprints. In feature extraction (PCA and LDA), five palmprint images of each person are used for training and the rest 1483 palmprint images are used for test.

In experiments, the number of genuine points is set to be 31; for each genuine point, 20 chaff points are generated for fuzzification using proposed chaff point generating algorithm. And one symbol error is set to be corrected by ECC.

### 4.2. Accuracy Experiments

Similar to biometric verification system, receiver operating characteristic (ROC) curve (which includes two kinds of error rates, that is, the false accept rate (FAR) and the false reject rate (FRR)) and equal error rate (EER) (when FAR = FRR) are used to evaluate the accuracy of proposed hybrid system. ROC curves are obtained by varying the controlling distance between chaff vectors and genuine vectors. EER curves are obtained under different dimensionality of projected feature vectors.

In the random projection module of proposed hybrid system, random matrices and biometric templates are needed for feature transformations, so it is a two-factor scheme. Three different scenarios, that is, stolen-key, stolen biometrics, and both legitimate cases, should be considered.

For the stolen-key case, the impostor will use genuine projection matrices and impostor biometrics for vault unlocking. This is equal to user-independent (UI) scenario; that is, different users use the same projection matrices for vault unlocking, which characterizes the system accuracy when user-independent transformations are used. For the stolen-biometrics scenario, random generated projection matrices and genuine biometrics are used for vault unlocking. In both legitimate cases, different user uses different projection matrices for vault locking and unlocking. This is a user-dependent (UD) scenario.

Let vault = Gen(*b*, *R*, *S*), where “Gen” represents vault generation algorithm, *b* represents biometric features used for vault generation, *R* represents projection matrices used for feature transformations, and *S* is the secrets to be protected by the vault. Given genuine query biometrics *b*_*L*_ and legal query matrix *R*_*L*_, if *S* ≠ Unlock(vault, *b*_*L*_, *R*_*L*_), where “Unlock” represents vault unlocking algorithm, this is false reject case. Given impostor query biometrics *b*_*I*_ and impostor query matrix *R*_*I*_, if *S* = Unlock(vault, *b*_*I*_, *R*_*I*_), where “Unlock” represents vault unlocking algorithm, this is false accept case.

**Figure 4:**
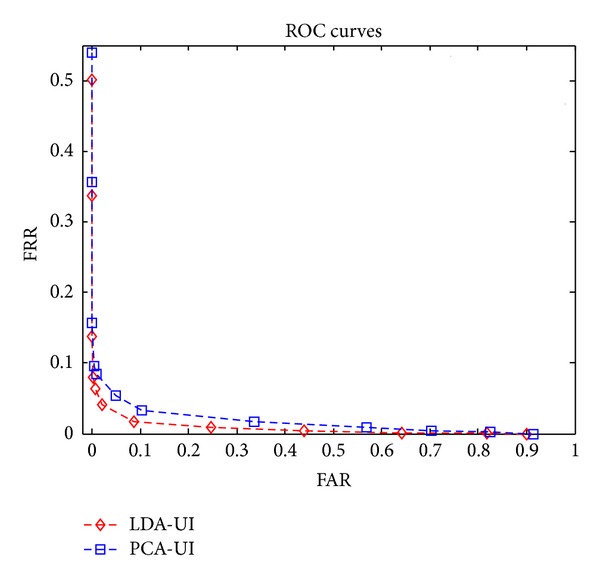
ROC curves in user-independent scenario.

Figure 4 shows the ROC curves in user-independent scenario. The dimensionality of genuine vector is 100. The LDA feature outperforms PCA feature because the random projection can only preserve the discriminability of features but cannot enhance that in user independent case. And LDA features have better discriminability than PCA features, as we know. The user-dependent scenario is not shown in Figure 4; in fact, FRR decreases by enlarging the distances between chaff vectors and genuine vectors and vice versa, but the FAR remains at zero in experiments.

**Figure 5:**
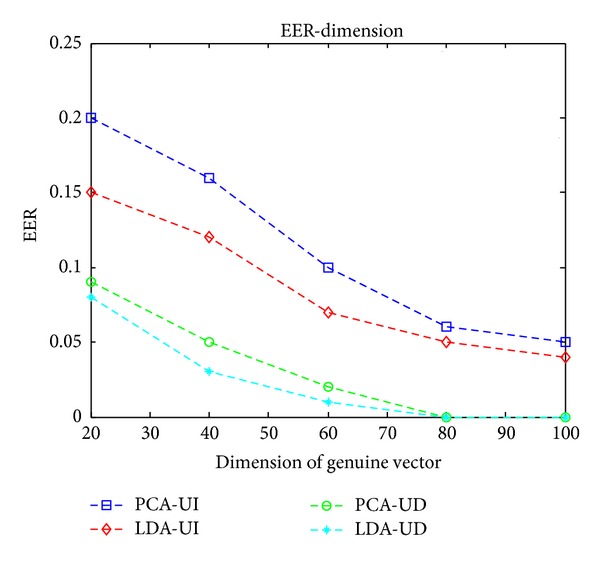
EER curves.

EER curves in Figure 5 are obtained by varying dimensionality of projected vectors. In user independent case, the EER decreases as the dimension increases, but no zero EER is obtained. For user-dependent scenario, the EER decreases to zero when dimensionality is equal or greater than 80. The zero EER of hybrid system benefits from the random projection module, in which user-dependent projection matrices enhance the discriminability of transformed biometric features.

### 4.3. Changeability Experiments

The changeability of proposed hybrid scheme is provided by the random projection module, where different enrolling features can be generated for different applications by applying random projection with different projection matrices.

Let vault = Gen(*b*_*i*_, *R*_*i*_, *S*), where *b*_*i*_ is the enrolled biometric features and *R*_*i*_ is the enrolled projection matrix. Using random generated projection matrices *R*_*j*_ and genuine biometric features *b*_*j*_ to unlock the vault, if *S* = Unlock(vault, *b*_*j*_, *R*_*j*_), this is the false accept case, the obtained FAR is used to measure the changeability of proposed scheme.

In experiments, each test palmprint feature vector is paired with five groups of randomly generated matrices to unlock the corresponding vault. There are 1483 test palmprints; 7415 times experiments are performed totally.

**Figure 6:**
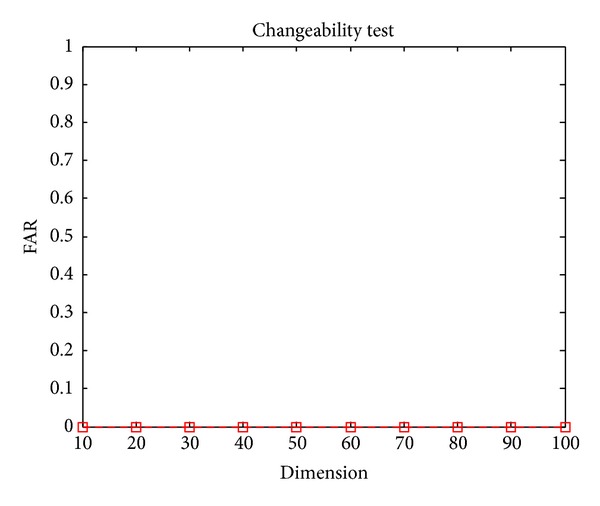
Changeability.

The experimental results are shown in Figure 6. It can be seen that with different projection dimension, the FAR is always zero, which means that the proposed hybrid algorithm can provide strong changeability.

### 4.4. Security Experiments

According to the theoretical analysis of security in Section 3.3, in this section we consider the quantized security bits in the worst case (i.e., the attacker has known projection matrices and has impostor biometrics) based on the experimental parameters.

In our experiments, the number of genuine points *t* = 31. In fuzzification, 20 chaff points are added around each genuine point, so the total number of points *r* = 651. And one symbol error can be corrected by ECC; that is, *k* = 1. Substituting these parameters into (20), the obtained security bits are 131.77 bits, which is higher compared to those typically reported in the literature [9, 10, 20–22].

**Figure 7:**
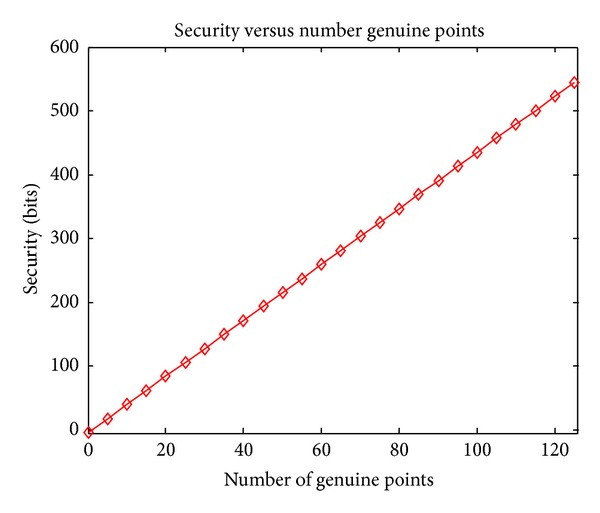
Security by varying the number of genuine points.

**Figure 8:**
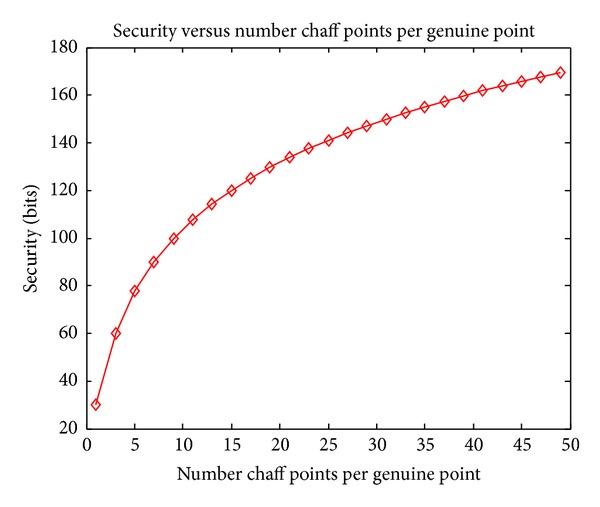
Security by varying the number of chaff points around each genuine point.

**Figure 9:**
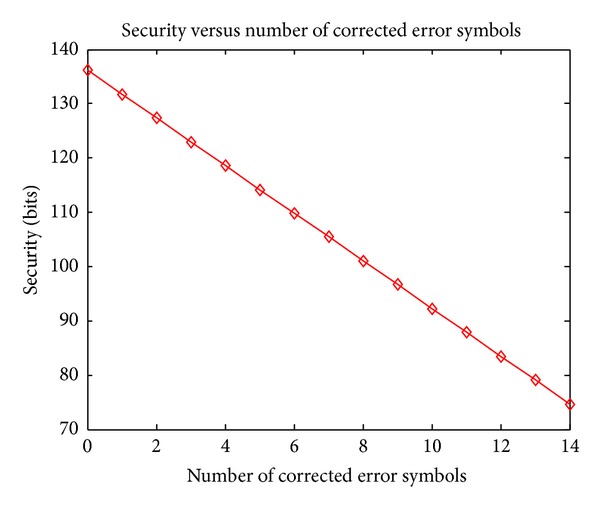
Security by varying the number of error symbols that can be corrected by ECC.

Figures 7–9 show how the security bits change by varying parameters *r*, *t*, and *k*. From Figure 7 we can see that the security bits increase rapidly by increasing the number of genuine points *t*. From Figure 8 we can see that the security also increases by adding more chaff points around each genuine points, but the growth rate decreases when the number of chaff points increases. From Figure 8 we can see that with the increasing of corrected number of corrected error symbols, the security decreases; this indicates the tradeoff between accuracy and security; that is, correcting more symbol errors can decrease the FRR of system, but the security also decreases and vice versa.

## 5. Conclusions

To better satisfy accuracy, changeability, and security requirements for biometric template protection, in this paper, a hybrid approach for protecting real-valued palmprint feature vectors has been proposed. The proposed hybrid approach includes two modules: random projection and fuzzy vault scheme. A heterogeneous space was proposed for fuzzy vault to enhance the intraclass variant tolerating ability and the cryptographic key can be bound as long as needed. To improve the security of fuzzy vault in heterogeneous space, a chaff point generation method was also proposed.

Theoretical analyses from accuracy, changeability, and security perspectives were presented. For accuracy analysis, orthogonal projection and nonorthogonal projection were considered. For changeability analysis, statistical properties of projected feature vector were obtained using same projection matrices and different projection matrices have shown that higher dimension of projected feature vectors provides stronger cancelability. For security analysis, we considered four different scenarios that the attacker knows different information.

Experiments based on HA-BJTU palmprint database have given concrete data to support the proposed hybrid approach well in the view of accuracy, changeability, and security.
